# Simulation-Based Evaluation of Three Methods for Local Ancestry Deconvolution of Non-model Crop Species Genomes

**DOI:** 10.1534/g3.119.400873

**Published:** 2019-12-20

**Authors:** Aurélien Cottin, Benjamin Penaud, Jean-Christophe Glaszmann, Nabila Yahiaoui, Mathieu Gautier

**Affiliations:** * CIRAD, UMR AGAP, F-34398 Montpellier, France,; † AGAP, Univ. Montpellier, CIRAD, INRAE, Montpellier SupAgro, Montpellier, France, and; ‡ INRAE, UMR CBGP, F-34988 Montferrier-sur-Lez Cedex, France

**Keywords:** local ancestry inference, crops, admixture, simulation analysis

## Abstract

Hybridizations between species and subspecies represented major steps in the history of many crop species. Such events generally lead to genomes with mosaic patterns of chromosomal segments of various origins that may be assessed by local ancestry inference methods. However, these methods have mainly been developed in the context of human population genetics with implicit assumptions that may not always fit plant models. The purpose of this study was to evaluate the suitability of three state-of-the-art inference methods (SABER, ELAI and WINPOP) for local ancestry inference under scenarios that can be encountered in plant species. For this, we developed an R package to simulate genotyping data under such scenarios. The tested inference methods performed similarly well as far as representatives of source populations were available. As expected, the higher the level of differentiation between ancestral source populations and the lower the number of generations since admixture, the more accurate were the results. Interestingly, the accuracy of the methods was only marginally affected by i) the number of ancestries (up to six tested); ii) the sample design (*i.e.*, unbalanced representation of source populations); and iii) the reproduction mode (*e.g.*, selfing, vegetative propagation). If a source population was not represented in the data set, no bias was observed in inference accuracy for regions originating from represented sources and regions from the missing source were assigned differently depending on the methods. Overall, the selected ancestry inference methods may be used for crop plant analysis if all ancestral sources are known.

Inter-(sub)-specific hybridizations have shaped the genomes of many crop species as for example in wheat (El Baidouri *et al.* 2017), rice (Zhao *et al.* 2010), citrus (Wu *et al.* 2014, 2018), banana (Perrier *et al.* 2011) or apple (Cornille *et al.* 2012). They can be a consequence of germplasm transport by humans bringing together plants from related but differentiated species, subspecies or populations, or of gene flow between cultivated plants and neighboring wild relatives (*e.g.*, Semon *et al.* 2005; Perrier *et al.* 2011). At the genome level, such admixture events can result in a mosaic pattern of chromosomal segments of various origins. The complexity of the mosaic will depend on the demographic characteristics of the populations (*e.g.*, number of source ancestries and the timing of admixture events). As already demonstrated in other species such as humans, characterizing the genome mosaic may in turn provide valuable insights into the genetic history of populations (*e.g.*, Moreno-Estrada *et al.* 2013; Hellenthal *et al.* 2014), and in the context of domesticated plant species, it may lead to a better understanding of crop domestication and diversification history. It might also help identifying the origin of introgressed variants underlying agricultural traits of interest (Burgarella *et al.* 2019) and possibly support breeding strategies to produce improved hybrids.

Over the past twenty years, the development of high-density genotyping and sequencing technologies has promoted the development of accurate approaches to infer genetic ancestry of individuals based on genotyping data. Historically, the first proposed methods aimed at characterizing individual ancestries on a genome-wide scale by estimating the relative contributions of a given number of underlying ancestries. The most popular of these methods are based on unsupervised clustering approaches as introduced by Pritchard *et al.* (2000) in the Structure software, where clusters were interpreted as proxies for ancestries. An extension of the Pritchard’s work by Falush *et al.* (2003), further allowed to perform local ancestry inference (LAI), *i.e.*, to infer the ancestral origin at a local chromosome scale in individual genomes. Since then and as reviewed in Geza *et al.* (2019), more than 20 LAI methods have been published extending, in particular, this pioneering work by scaling up to high throughput genotyping data or by leveraging phased data for more accurate inferences.

Most LAI approaches have been developed in the context of human genetics studies for which their properties have been extensively characterized (Liu *et al.* 2013; Padhukasahasram 2014; Hui *et al.* 2017; Geza *et al.* 2019). Human studies relying on LAI approaches usually aim at assessing admixture between two or three populations and may benefit from a rich amount of genetics resources (The International HapMap Consortium 2005; The Wellcome Trust Case Control Consortium 2007) with, in particular, dense haplotype data for reference populations and/or admixed samples. For plant species, large-scale sequencing or genotyping resources are also increasingly available for some crops such as rice (The 3,000 rice genomes project 2014) or barley (Milner *et al.* 2019). However, for many other species of interest such resources remain scarce which implies that haplotype data may not yet be fully accessible, particularly for non-autogamous species. In addition, ancestries may be multiple as exemplified by the cacao tree (*Theobroma cacao*) germplasm which is composed of 10 major genetically differentiated groups with up to six-way admixed individuals (Cornejo *et al.* 2018) or pineapple (*Ananas comosus*) with up to four-way admixed individuals between cultivar groups and varieties (Chen *et al.* 2019). Moreover, populations representative of contributing ancestries may be unavailable or represented by only a few individuals. To that respect, the case for banana is particularly illustrative since hybridization events involving well-differentiated *Musa acuminata* subspecies are predicted to be involved in the formation of some major cultivars (Perrier *et al.* 2009, 2011). Yet, some of these subspecies are represented only by a few individuals (Christelová *et al.* 2017) or some contributors may not be represented in available germplasm (Sardos *et al.* 2016). Moreover, it remains unclear how some features, regarding reproduction modes (*e.g.*, selfing or vegetative reproduction) that may be encountered in plant models, may affect the performance of LAI methods. Hence, in fruit crops such as citruses or banana, individuals resulting from inter(sub)specific hybridizations were further multiplied by vegetative propagation (also termed clonal propagation). Thus, they do not form a population but rather a collection of individuals sometimes of different origins with ancestry mosaics of relatively large blocs depending on the number of sexual generations they may have undergone. Datasets of vegetatively propagated individuals may thus be heterogeneous in terms of ancestry structure and in terms of time in generations since admixture events. The number of sexual generations since admixture is a parameter that is often required by LAI programs (Geza *et al.* 2019) and it can be difficult to correctly estimate it for plants that have been vegetatively propagated sometimes since hundreds of years. On the other hand, high selfing rates result in increased levels of homozygosity and generally in reduced diversity levels compared to outcrossing species (Brandvain *et al.* 2013; Barrett *et al.* 2014) while introducing additional levels of structuring of haplotype diversity when selfing and outcrossing populations are analyzed together. Finally, polyploidy that is a feature of many crop plants (*e.g.*, wheat, sugarcane, potato, and major banana cultivars) is still a complex case to handle for LAI as genotypes are difficult to infer.

The purpose of this study was to evaluate the accuracy of LAI approaches to perform ancestry deconvolution, based on genotyping data simulated under scenarios that can be representative of plant species models. Given the lack of available methods dealing with polyploids, we only considered diploid individuals. Among the 22 LAI approaches recently reviewed by Geza *et al.* (2019), we chose to evaluate three methods – SABER (Tang *et al.* 2006); ELAI (Guan 2014) and WINPOP (Paşaniuc *et al.* 2009) because they do not require prior phasing of the data and they could cope with more than two ancestries. We developed an R package to perform simulations and we focused our evaluation on the influence of i) the level of divergence between the source populations; ii) the number of generations since admixture for the admixed populations; iii) the number of contributing ancestries and their representation in the analyzed data sets; and iv) the mode of reproduction such as selfing (in a source representative population) or vegetative propagation (in the admixed population).

## Material and Methods

### Simulation tool

**Figure 1:**
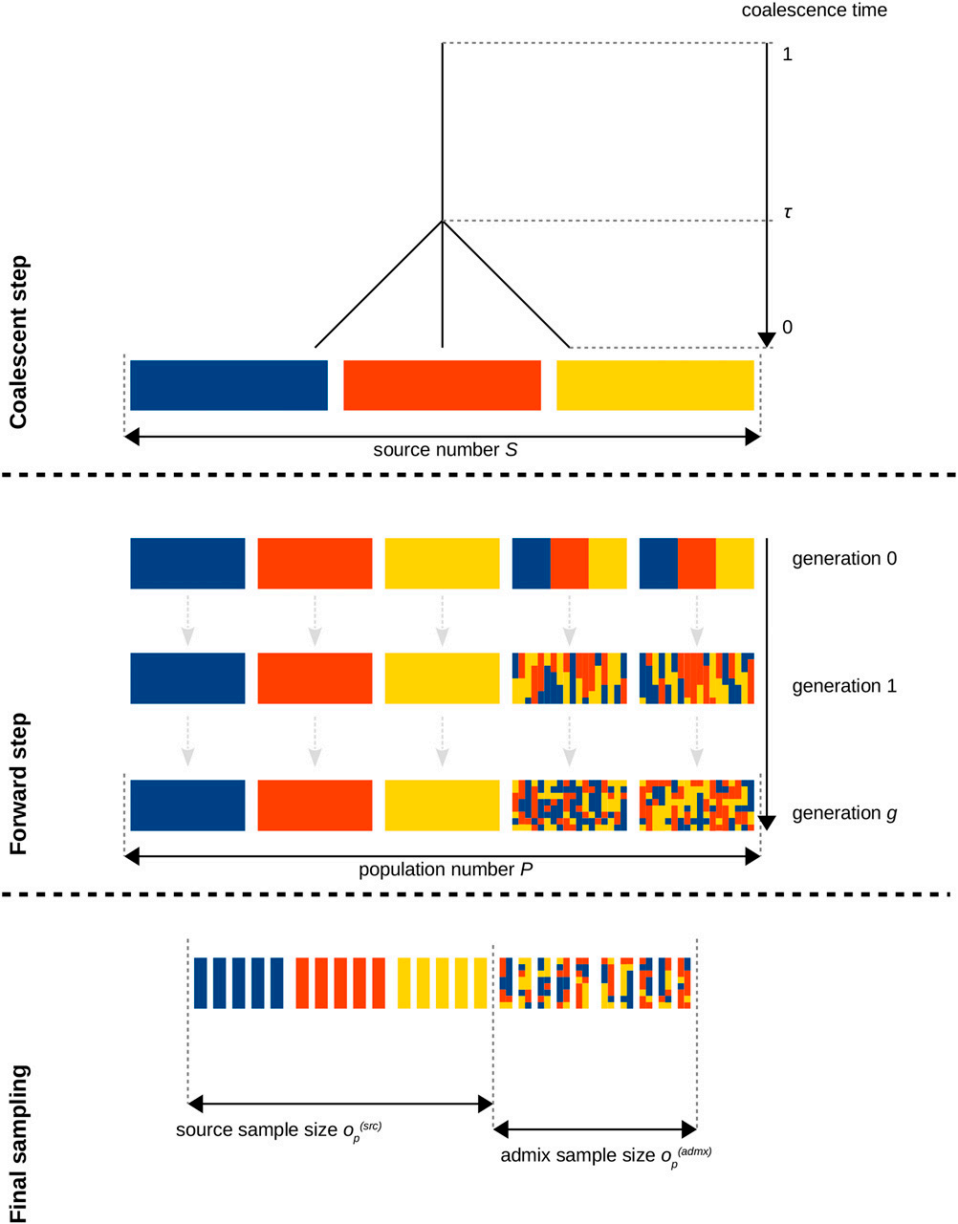
Overview of the admixture simulation process with *plmgg*. The coalescent step produces *S* source populations (here, three sources are represented in blue, red and yellow) that differentiated at τ. In the forward step, source-representative populations and admixed populations are generated from sampling of the source populations. Then, each population follows for a number of generations g, a user-defined reproduction process that allows to select and combine reproduction modes (within population random mating, across population random mating, selfing, vegetative reproduction). In the last step, a sampling is performed on each population of the forward step to generate a data set for analysis.

We developed an R package (named *plmgg* for plant-like mosaic genome generator) to simulate individual chromosome-wide genotyping data from an arbitrary number of populations P deriving from S differentiated source populations under scenarios that may include hybridization events and modes of reproduction representative of plant model evolution (*i.e.*, selfing or vegetative propagation). The simulation approach is depicted in Figure 1 and consisted of the three following successive steps i) coalescent simulation of a sample of founder chromosomes from S differentiated source populations; ii) forward in-time simulation of P populations deriving from the S source populations with complex demographic scenarios involving various modes of reproduction and admixture; and iii) sampling of individuals from the P populations to generate the genotyping data sets. For coalescent simulations, we relied on the *scrm* algorithm (Staab *et al.* 2015) implemented in the R package *coala* (Staab and Metzler 2016) to simulate $n_{S}^{\left(h\right)}=\sum_{s=1}^{S}n_{s}^{\left(h\right)}$ founder chromosomes (*i.e.*, haploid (h) individuals) from S predefined source populations (where $n_{s}^{\left(h\right)}$ is the number of chromosomes from source population s). Sources were assumed to derive from a single ancestral population under a pure-drift model of divergence with a star-shaped history. The divergence scenario of the source populations was specified with three parameters: i) the divergence time τ measured in units of $4N_{e}$ (*i.e.*, $\tau =\frac{t}{4N_{e}}$ where t is the number of generations since the ancestral population and $N_{e}$ is the haploid effective source population size assumed to be the same for the S source populations); ii) the scaled mutation rate $\theta =4N_{e}µ$ (where µ is the mutation rate per site and per generation); and iii) the scaled recombination rate $\rho =4N_{e}r$ (where r is the recombination rate per site and per generation). For the purpose of this study, τ was varied to control the level of differentiation among the source populations (see below) while both θ and ρ were set equal to 10^−4^ (as obtained for instance if one assumes $µ=2.5\times 10^{-8}$ mutation and $r=2.5\times 10^{-8}$ recombination per site and per generation in a population of haploid size $N_{e}=10^{3}$).

In the second simulation step, $n_{P}=\sum_{p=1}^{P}n_{p}$ diploid individuals belonging to P populations (where $n_{p}$ is the number of diploid individuals from population p) were first generated by randomly sampling two chromosomes (without recombination and with replacement) among the $n_{S}^{\left(h\right)}$ founder ones according to the S pre-defined source contributions (in a P×S ancestry proportion matrix). The $n_{P}$ individuals were further reproduced over G generations, in a forward-in-time process, by specifying for each generation g (in a P×G matrix) the proportions of the four following possible population-specific modes of reproduction: i) within population random mating; ii) across population random mating; iii) selfing; and iv) vegetative reproduction (consisting of randomly reproducing an individual’s chromosome pair from one generation to the next). For sexual reproduction events (*i.e.*, random mating and selfing), parental gametes were generated by randomly distributing one crossing-over between the two parental chromosomes which amounts to assume a 1 Morgan length chromosome map. In addition, mutations that only affected existing variant positions (switch to the alternate SNP allele) were introduced at each generation at the rate µ defined above (whatever the reproduction mode). In other words, no new segregating sites appeared after the initial coalescent phase of the simulation (first simulation step described above).

In the third and last step of the simulation, $o_{P}=\sum_{p=1}^{P}o_{p}$ diploid individuals belonging to the P populations were sampled to generate the data set to be analyzed (where $o_{p}$ represents the number of genotyped diploid individuals from population p that are randomly sampled with replacement from the corresponding $n_{p}$ individuals available at generation G). After filtering out monomorphic SNPs, the simulation output consists of i) the genotyping data set in vcf (Danecek *et al.* 2011) and *plink* ped (Purcell *et al.* 2007); ii) the true local ancestry for each individual at each SNP position which may be displayed with plotting functions; and iii) summary statistics including pairwise population $F_{ST}$ (Weir and Cockerham 1984), population heterozygosities and ancestry block sizes.

### Simulated scenarios

Six scenarios detailed in Table 1, each replicated 50 times, were considered for this study. The number of founder chromosomes was set to $n_{s}^{\left(h\right)}=300$ for each source population (thereby mimicking bottlenecks involved by the domestication process from a small number of wild relatives) and the number of diploid individuals was set to $n_{p}=150$ for all the populations (*i.e.*, the source and admixed populations). Forward simulations were run for G=50 generations maintaining *S* non-admixed populations as source population proxies and two populations originating from an admixture event between three or more ancestries that occurred from $t_{adm}=5$ to 50 generations ago. Unless otherwise stated, the sampled data set consisted of $o_{p}=20$ diploid individuals for each ancestry representative population and $o_{p}=40$ individuals for each admixed population. The scenarios were split into three groups to investigate the effect of i) the ancestry representative sample size; ii) the number of sources; and iii) the reproduction modes. First, the DiffGenSam scenarios (Table 1) aimed at evaluating the impact of the amount of differentiation between S=3 source populations (with τ varying from 0.05 to 0.40); the number of generations since admixture for the two admixed populations (from $t_{adm}=5$ to 50); and the sample size (from $o_{p}=5$ to 40) of each of the three ancestry-representative populations. Five other scenarios were subsequently considered to address specific points while setting τ=0.20 and $t_{adm}=50$ (Table 1). The SamBal scenario aimed at evaluating the impact of unbalanced sample sizes among three ancestry representative populations (*i.e.*, two with 20 sampled individuals and the remaining with 2 to 20 sampled individuals). We also considered two scenarios to address the impact of the number of source populations (SrcNum with S=3 to 6 source populations equally contributing to the admixed populations) or the presence of a non-sampled source population contributing to the admixed individuals (SrcMiss). In the latter case, S=4 source populations were simulated, but only three of them had representatives in the final data set. The contribution of the “missing” source population to the admixed populations varied from 0.05 to 0.15, the three other sources having equal contributions. Finally, the SrcSelf and AdmxVegProp scenarios aimed at investigating the impact of alternative modes of reproduction. In the SrcSelf scenario, we assumed that one of the three source populations was reproducing with a selfing rate varying from 0 (*i.e.*, no selfing) to 0.99. The AdmxVegProp scenario modeled 10 admixed populations (with $n_{p}=100$ and $o_{p}=10$ for each admixed population) that switched from exclusive within population random mating to exclusive vegetative propagation $t_{veg}$ generations ago, with $t_{veg}$ varying from 0 (*i.e.*, no vegetative propagation) to 45 for the 10 populations. Note that the realized number of SNPs (after filtering steps) in the different simulated data sets ranged from $10^{4}$ to $2.7\times 10^{4}$ (File S1).

**Table 1:** Summary of simulation parameters

SIMULATION	τ	S	G	$P^{\left(src\right)}$	$P^{\left(admx\right)}$	$o_{p}^{\left(src\right)}$	$o_{p}^{\left(admx\right)}$	*Un*(%)	*Slf*(%)
DiffGenSam	0.05 - 0.40	3	5-50	3	2	5-40	40	0	0
SamBal	0.20	3	50	3	2	2-20*^a^*	40	0	0
SrcNum	0.20	3-6	50	3-6	2	5	40	0	0
SrcMiss	0.20	4	50	3*^b^*	2	20	40	0-15	0
SrcSelf	0.20	3	50	3	2	20	40	0	0-99
AdmxVegProp	0.20	3	50	3	10	20	10	0	0

τ, differentiation time; S,number of sources; G, number of generations after the admixture event; $P^{\left(src\right)}$, numbers of source-representative populations; $P^{\left(admx\right)}$, number of admixed populations; $o_{p}^{\left(src\right)}$, sample size of source-representative populations; $o_{p}^{\left(admx\right)}$, sample size of admixed populations; *Un*, percentage of unknown source; *Slf*, percentage of selfing in the third source-representative population.

a 2-20 indicate the variation of $o_{p}^{\left(src\right)}$ on the third source-representative population, while the two others are fixed at $o_{p}^{\left(src\right)}$ = 20.

b $P^{\left(src\right)}$ here equals to S−1 to simulate a missing source-representative population.

### LAI methods

As mentioned in the introduction, we retained the three LAI methods respectively implemented in the programs SABER, WINPOP and ELAI, that do not require prior phasing of the data and that could cope with more than two ancestries. The two methods SABER (Tang *et al.* 2006) and ELAI (Guan 2014) rely on Hidden Markov Models (HMM) with an explicit modeling of LD, while WINPOP (Paşaniuc *et al.* 2009) relies on a model-based LD-free approach.

More precisely, SABER (Tang *et al.* 2006) extended the HMM by Falush *et al.* (2003), to account for background LD existing in ancestral populations by modeling the joint distribution of alleles from consecutive markers within each ancestral population. In addition, SABER allows modeling an arbitrary number of ancestral groups that may admix at different times estimated by a Likelihood Maximization algorithm (*saberML* function, here initialized with the simulated values). Each individual SNP-specific ancestry estimates were calculated as the posterior probability obtained with the forward-backward algorithm implemented in the *pipeline* function.

ELAI (Guan 2014) implements a two layers HMM to model two different scales of LD: the admixture LD (between alleles from different source populations) and a shorter ranged LD existing between alleles within each source population. This is achieved by introducing a local structuring of haplotypes into i) upper-layer clusters that represent different groups (interpreted as source populations); and ii) lower-layer clusters that represent group-specific haplotypes. We here set the number of upper clusters to the number *S* of simulated sources; the number of lower clusters to 5S as recommended; and the time since admixture (also required by ELAI) to the corresponding simulated one. Model fitting was carried out with the default Expectation-Maximization (EM) algorithm.

WINPOP, included in LAMP >=2.3 (Sankararaman *et al.* 2008), is a model-based LD-free method that focuses on ancestry informative markers (AIM) to assign local haplotype blocks to their originating source populations (Paşaniuc *et al.* 2009). WINPOP works with variable-size overlapping windows along the chromosomes, and uses a clustering method to assign ancestries in each window, based on estimates of global ancestry proportions. WINPOP was used with the simulated recombination rate and default parameters for the configuration files including a LD pruning cutoff of r^2^ = 0.1 and a fraction of sliding window overlap of 20%.

Both WINPOP and SABER required estimates of global ancestry proportion. These were obtained by running the default unsupervised hierarchical clustering algorithm implemented in the ADMIXTURE software (Alexander *et al.* 2009) setting the number of clusters to *S*, the number of simulated source populations.

### Evaluation of the performance of LAI methods

To evaluate the performance of the LAI methods, we defined an accuracy metric α to quantify the overall differences between simulated and inferred local ancestries. Let $z_{s,m}^{\left(i\right)}$ represent the simulated proportion of ancestry s(s=1,…, S) at SNP position m(m=1,…,M); M being the number of SNPs) for individual i ($z_{s,m}^{\left(i\right)}=$0, 0.5 or 1 since individuals are diploid). Similarly, let $x_{s,m}^{\left(i\right)}$ represent the inferred proportion of ancestry. The SNP-specific accuracy for individual i over the S ancestries was then defined as $\alpha_{m}^{\left(i\right)}=1-\sum_{s=1}^{S}\frac{\left|z_{s,m}^{\left(i\right)}-x_{s,m}^{\left(i\right)}\right|}{2}$. Note that $\sum_{s=1}^{S}\left|z_{s,m}^{\left(i\right)}-x_{s,m}^{\left(i\right)}\right|$ is bound between 0 (when $z_{s,m}^{\left(i\right)}=x_{s,m}^{\left(i\right)}$ for all s) and 2 (when $z_{s,m}^{\left(i\right)}=0$ or $x_{s,m}^{\left(i\right)}=0$ for all s since $\sum_{s=1}^{S}z_{s,m}^{\left(i\right)}=1$ and $\sum_{s=1}^{S}x_{s,m}^{\left(i\right)}=1$), hence the division of this sum by 2 in the definition of the accuracy $\alpha_{m}^{\left(i\right)}$ to keep $\alpha_{m}^{\left(i\right)}$ between 0 and 1. The overall accuracy metric α was defined as the average of $\alpha_{m}^{\left(i\right)}$ over the M markers and the individuals belonging to admixed populations (*i.e.*, excluding individuals belonging to source representative populations). For the particular case of SrcMiss simulations (Table 1) in which one source representative population was missing, SNP positions with the corresponding missing ancestry were excluded from the computation of α. According to our definition, α always lies between 0 and 1 (the higher the α value, the more accurate the inference). For calibration purposes, we also computed a minimal value of α as would be obtained by randomly inferred local ancestries under the assumptions of equal contribution of the sources (*i.e.*, setting $x_{s,m}^{\left(i\right)}=1/S$ for all s). Alternative metrics, such as the coefficient of determination (*i.e.*, sample correlation coefficient between the inferred and true local ancestries) or mean square errors were also evaluated but were not presented since they lead to the same conclusions regarding the ranking of LAI methods.

We finally evaluated computational efficiency of the different LAI programs by recording for each run of analysis on our computer grid, both the memory usage and the system computing time (*max_vmem* and *ru_wallclock*, respectively) available from the Sun Grid Engine user notification.

### Data availability

The R package *plmgg* is available at https://gitlab.southgreen.fr/acottin/plmgg. Scripts used to run simulations, LAI programs and inference comparison are available at https://gitlab.southgreen.fr/acottin/lai-comparison. Supplemental material available at figshare: https://doi.org/10.25387/g3.10266149.

## Results

### Source differentiation and number of generations Since admixture

**Figure 2:**
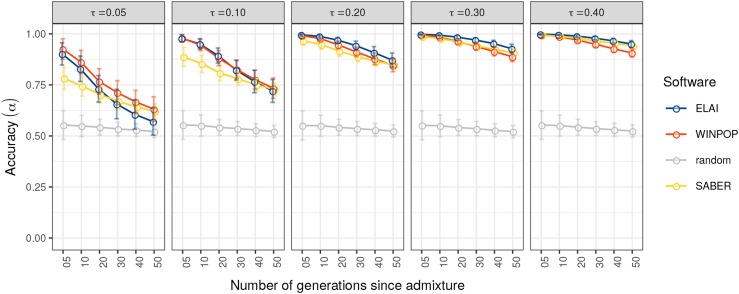
Accuracy of LAI methods with varying levels of differentiation and number of generations (DiffGenSam simulation). The accuracy (α) of the LAI methods (y-axis) is plotted for different levels of differentiation that vary from 0.05 to 0.4 (vertical tiles) and a number of generations after admixture that varies from 5 to 50 (x-axis). The sample size is set to 20 for the sources and the admixed populations. Each dot is the mean value of 50 repetitions of each simulation. Error bars indicate the standard deviation. ELAI, WINPOP and SABER scores are plotted in blue, red and yellow, respectively. Accuracy of random inference (proportion of ancestry fixed at 1/3) is plotted in gray.

The impact of the level of differentiation among the sources and the number of generations since admixture on the performance of the three LAI methods was assessed with the DiffGenSam scenarios (Table 1). The analysis of the generated data sets showed that both the level of differentiation among sources and the number of generations since admixture had a strong impact on the performance of LAI methods (Figure 2, File S2). Indeed, the accuracy α decreased with an increasing number of generations after admixture (*i.e.*, when ancestry block sizes became smaller) and with decreasing levels of differentiation between source populations (Figure 2). Although, the three evaluated LAI approaches performed overall similarly, at the lowest levels of differentiation (τ≤0.10), ELAI and WINPOP were more accurate than SABER for more recent admixture events ($t_{adm}\leq 20$) (Figure 2, File S2). In the most favorable situations of high differentiation among the source populations (*i.e.*, τ≥0.3), the accuracy α tended toward 1 (*i.e.*, no error) with decreasing time since admixture for all the three LAI methods.

### Number of individuals from the source representative populations

**Figure 3:**
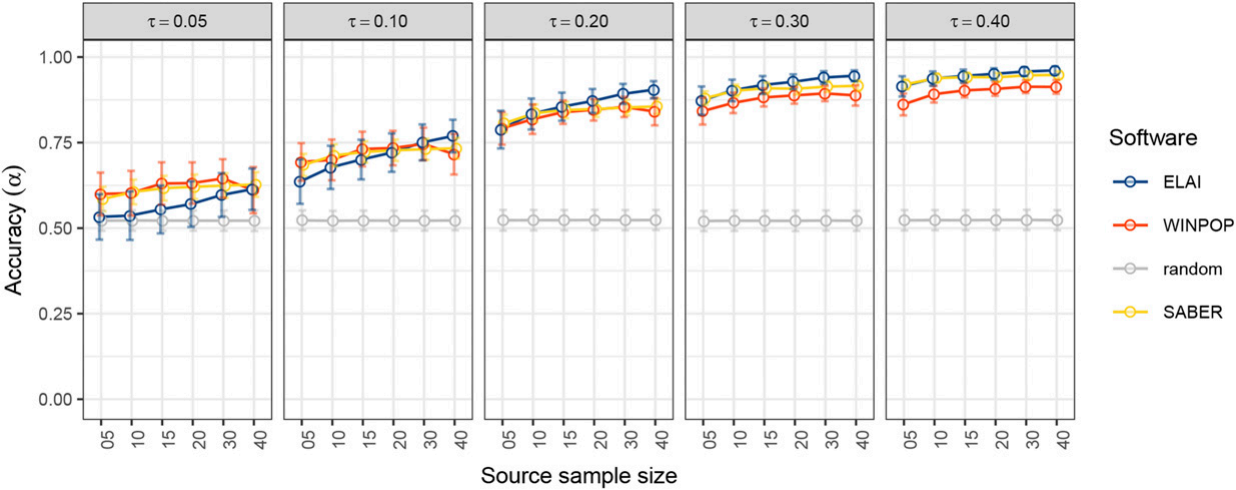
Accuracy of LAI methods with varying levels of differentiation and source-representative sample size (DiffGenSam simulation). The accuracy (α) of the LAI methods (y-axis) is plotted for different levels of differentiation that vary from 0.05 to 0.4 (vertical tiles) and the size of source-representative sample that varies from 5 to 40 individuals (x-axis). The source sample size is set to 20. Each dot is the mean value of 50 repetitions of each simulation. Error bars indicate the standard deviation. ELAI, WINPOP and SABER scores are plotted in blue, red and yellow, respectively. Accuracy of random inference (proportion of ancestry fixed at 1/3) is plotted in gray.

The impact of the number of sampled representative individuals for each of the three source populations was also evaluated within the DiffGenSam scenarios (Table 1). As shown in Figure 3, for a given time since admixture (here $t_{adm}=50$, see Figure S1 and File S2 for alternative $t_{adm}$ values) decreasing the number of individuals representative of the source populations (*e.g.*, from $o_{p}^{\left(s\right)}=20$ as in Figure 2 to $o_{p}^{\left(s\right)}=5$) had a higher impact on accuracy for ELAI compared to WINPOP and SABER. Conversely, except for the highest level of differentiation among source populations, increasing the number of source representative individuals improved ELAI performances.

We evaluated the robustness of the three LAI methods to unbalanced sample sizes of source-representative populations by analyzing data sets simulated under the SamBal scenarios where the number of samples was reduced for one of the three sources (Figure S2, File S3). The accuracy of ELAI was lower than both the WINPOP and SABER when sampling was reduced for the third source (*e.g.*, for 2 representatives instead of 20, accuracy of 0.720 for ELAI *vs.* 0.815 for WINPOP and 0.806 for SABER, File S3) but it increased when sampling was more balanced reaching accuracy of 0.870 for a completely balanced setting. For WINPOP and SABER the accuracy was only marginally improved, reaching up to 0.850.

According to the results above, to allow better discrimination of the LAI methods in relatively challenging conditions, we chose to perform the remaining evaluations with a number of generations after admixture set to 50, a level of differentiation among sources of τ=0.2 and 20 individuals per source representative population.

### Number of source populations and absence of source representative individuals

**Figure 4:**
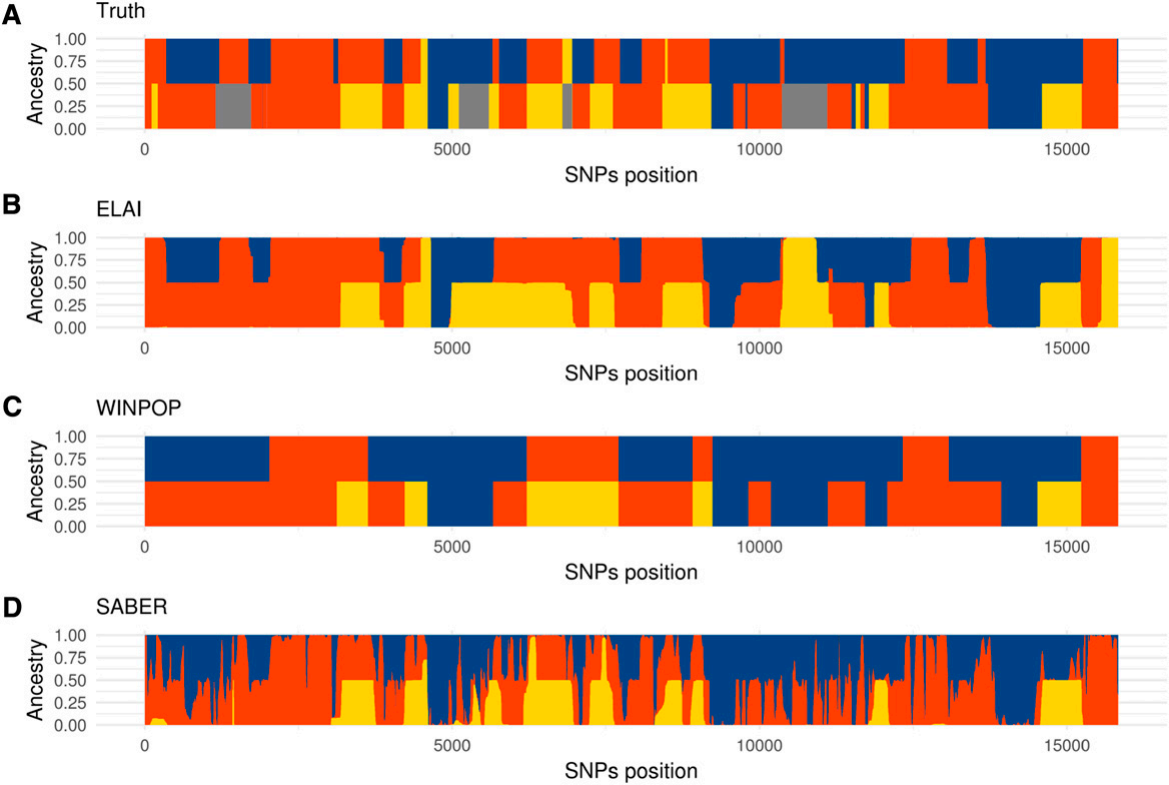
LAI results for a simulated admixed individual of the SrcMiss simulation. The x-axis indicates simulated SNPs positions and the y-axis represents the stacked ancestry proportions (between 0 and 1). (**A**) represents the true ancestries. (**B**), (**C**) and (**D**) represent the inference from ELAI, WINPOP and SABER, respectively. The three known sources are shown in blue, red and yellow and the unknown source in gray.

With the SrcNum scenarios (Table 1), data sets were simulated for admixture events involving up to six source populations. The analysis of LAI results showed that the accuracy decreased with increasing numbers of sources for all three evaluated LAI approaches (Table 2). However, the magnitude of decrease in accuracy from S=3 to S=6 source populations remained moderate with rates equal to 2.7%, 8.7% and 11% for ELAI, WINPOP and SABER respectively (to be compared with the 45% decrease observed with the random inference) (Table 2). We further assessed the impact of the absence of individuals from one out of four source representative populations using data sets simulated under the SrcMiss scenario (Table 1). Different proportions of this unrepresented source to admixed populations were tested (5, 10 and 15%) and accuracy was measured by excluding regions contributed by the missing source population. As shown in Table 3, the accuracy for all methods was stable in regions without the unknown ancestry, whatever the global proportions (at a data set level) of unknown ancestry. This suggested that the absence of individuals from a source representative population in the analyzed data sets did not introduce biases in inferring local ancestries of the represented source populations. Visual inspection of local ancestries inferred in regions containing the missing ancestry did not reveal any particular pattern (*e.g.*, like a higher switching rate among the other represented ancestries). As an example, Figure 4 shows the inferred local ancestry mosaic of one individual from a simulated data set with a 10% contribution of the unrepresented source population. In general, the chromosomal regions originating from the missing source population tended to be assigned to different represented ancestries, the assignation also varying according to the LAI method used.

**Table 2:** Accuracy of LAI methods with varying number of source populations (SrcNum scenario)

S	ELAI	WINPOP	SABER	Random
3	0.788 ± 1.7 10^−3^ (0.055)	0.792 ± 1.5 10^−3^ (0.048)	0.806 ± 0.8 10^−3^ (0.026)	0.523 ± 0.9 10^−3^ (0.030)
4	0.765 ± 1.6 10^−3^ (0.053)	0.758 ± 1.4 10^−3^ (0.044)	0.768 ± 0.9 10^−3^ (0.030)	0.411 ± 0.7 10^−3^ (0.022)
5	0.758 ± 1.6 10^−3^ (0.053)	0.730 ± 1.5 10^−3^ (0.047)	0.737 ± 1.0 10^−3^ (0.032)	0.336 ± 0.6 10^−3^ (0.018)
6	0.767 ± 1.5 10^−3^ (0.050)	0.723 ± 1.5 10^−3^ (0.048)	0.717 ± 1.0 10^−3^ (0.032)	0.285 ± 0.5 10^−3^ (0.015)

Mean accuracy α, accuracy confidence interval (0.95) and accuracy standard deviation (sd) of ELAI, WINPOP and SABER on simulated data with variation on the number of source (S) from 3 to 6 are indicated. Simulations were conducted with 50 repetitions, τ=0.2, 50 generations after admixture and 20 individuals sampled from each population. Random inference (1/S for each ancestry) was evaluated like LAI methods.

**Table 3:** Accuracy of LAI methods with varying proportions of an unknown source population in admixed populations (SrcMiss scenario)

*Un*(%)	ELAI	WINPOP	SABER	RANDOM
0	0.869 ± 1.2 10^−3^ (0.038)	0.843 ± 1.0 10^−3^ (0.033)	0.846 ± 0.7 10^−3^ (0.023)	0.523 ± 0.9 10^−3^ (0.030)
5	0.863 ± 1.3 10^−3^ (0.042)	0.844 ± 1.1 10^−3^ (0.035)	0.846 ± 0.8 10^−3^ (0.024)	0.522 ± 1.0 10^−3^ (0.031)
10	0.860 ± 1.4 10^−3^ (0.044)	0.842 ± 1.2 10^−3^ (0.038)	0.844 ± 0.8 10^−3^ (0.026)	0.520 ± 1.0 10^−3^ (0.033)
15	0.855 ± 1.5 10^−3^ (0.047)	0.844 ± 1.2 10^−3^ (0.040)	0.844 ± 0.8 10^−3^ (0.026)	0.518 ± 1.0 10^−3^ (0.035)

Mean accuracy α, accuracy confidence interval (0.95) and accuracy standard deviation (sd) of ELAI, WINPOP and SABER on simulated data with different percentage of a fourth source population participating to the admixture event are indicated. Accuracy was computed after removal of the unknown population segments in the admixed individuals, to measure the impact on well represented segments. Simulations were conducted with 50 repetitions, τ=0.2, 50 generations after admixture and 20 individuals sampled from each population. Random inference (1/S for each ancestry) was evaluated like LAI methods.

### Selfing and vegetative propagation

Table 4 gives the accuracy of the different LAI approaches on data sets simulated under the SrcSelf simulation (Table 1) in which the third source representative population reproduced with a varying extent of selfing. For the three LAI approaches, increased proportions of selfing in the third source representative population resulted in a decrease of accuracy, to a small extent. Indeed the decrease in accuracy between rates of selfing of 0 and 99% was equal to 0.92%, 6.6% and 3.5% for ELAI, WINPOP and SABER, respectively.

**Table 4:** Accuracy of LAI methods with varying proportions of selfing in a source-representative population (SrcSelf simulation)

*Slf*(%)	ELAI	WINPOP	SABER	RANDOM
0	0.871 ± 1.1 10^−3^ (0.035)	0.846 ± 1.0 10^−3^ (0.031)	0.848 ± 0.7 10^−3^ (0.023)	0.524 ± 0.9 10^−3^ (0.030)
25	0.873 ± 1.1 10^−3^ (0.035)	0.836 ± 1.1 10^−3^ (0.037)	0.844 ± 0.7 10^−3^ (0.024)	0.523 ± 0.9 10^−3^ (0.029)
50	0.870 ± 1.2 10^−3^ (0.038)	0.831 ± 1.2 10^−3^ (0.038)	0.838 ± 0.8 10^−3^ (0.024)	0.524 ± 0.9 10^−3^ (0.030)
75	0.864 ± 1.2 10^−3^ (0.040)	0.810 ± 1.3 10^−3^ (0.042)	0.828 ± 0.8 10^−3^ (0.026)	0.523 ± 0.9 10^−3^ (0.029)
99	0.863 ± 1.2 10^−3^ (0.039)	0.790 ± 1.8 10^−3^ (0.058)	0.818 ± 1.0 10^−3^ (0.032)	0.522 ± 0.9 10^−3^ (0.029)

Mean accuracy α, accuracy confidence interval (0.95) and accuracy standard deviation (sd) of ELAI, WINPOP and SABER on simulated data with variation on selfing proportion in the third source-representative population are indicated. Simulations were conducted with 50 repetitions, τ=0.2, 50 generations after admixture and 20 individuals sampled from each population. Random inference (1/S for each ancestry) was evaluated like LAI methods.

**Figure 5:**
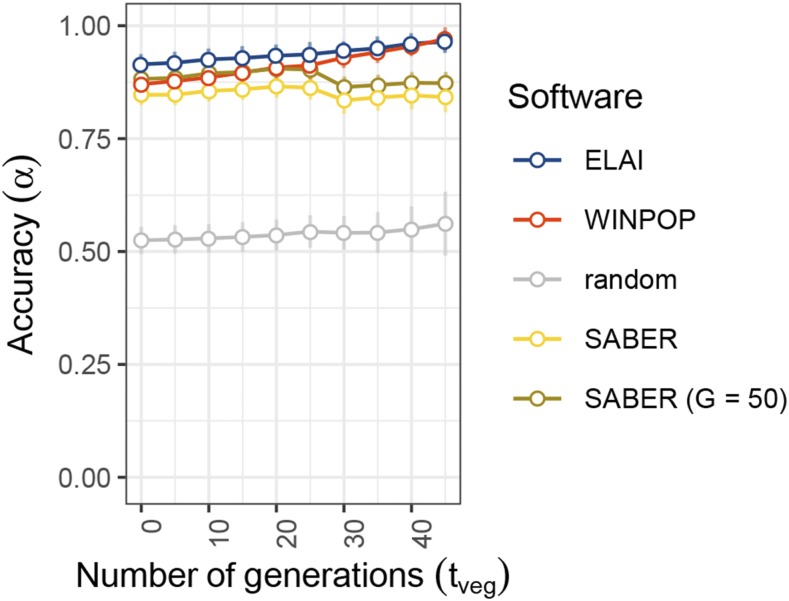
Accuracy of LAI methods with varying number of generations of vegetative propagation (AdmxVegProp simulation). The accuracy of the LAI methods (y-axis) is plotted for different numbers of generations of vegetative propagation after admixture ($t_{veg}$) that vary from 0 to 45 (x-axis). The source sample size is set to 20, the differentiation set to 0.2 and the total number of generations after the admixture event set to 50. Each dot is the mean value of 50 repetitions of each simulation. Error bars indicate the standard deviation. ELAI, WINPOP and SABER scores are plotted in blue, red and yellow, respectively. SABER score with fixed number of generations after admixture is plotted in darker yellow. Accuracy of random inference (proportion of ancestry fixed at 1/3) is plotted in gray.

Figure 5 plots the accuracies of LAI approaches estimated on data sets simulated under the AdmxVegProp scenarios (Table 1) consisting of individuals from three source representative populations and 10 admixed populations that switched to an exclusive vegetative propagation mode $t_{veg}$ generations ago ($t_{veg}$ varying from 0 to 45 for the different populations). Note that, the larger $t_{veg}$, the larger the ancestry block sizes (since the smaller the number of post-admixture recombinations). For both WINPOP and ELAI based inference, the accuracy increased for increasing values of $t_{veg}$ as expected given larger ancestry block sizes. However, the accuracy of SABER, being very similar for individuals with $t_{veg}=45$ and $t_{veg}=0$, was mostly not influenced by $t_{veg}$_,_ although a slight decrease was observed at $t_{veg}=30$. As this decrease appeared for higher numbers of generations of vegetative propagation, it may be linked to the fact that SABER performs its own estimation of time since admixture. To investigate this, a second run of SABER was performed without using the time since admixture estimation method (*saberML* function), but with a time since admixture fixed at $t_{adm}=50$ as for WINPOP and ELAI (Figure 5). SABER accuracy was found higher with this fixed number of generations but a decrease at $t_{veg}=30$ was still observed.

### Computational performances of LAI methods

**Figure 6:**
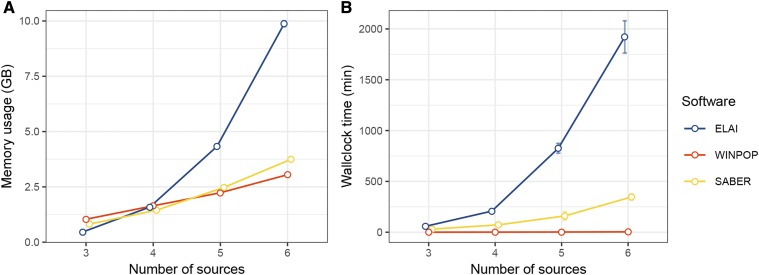
Memory usage and computation time of LAI methods with varying number of sources (SrcNum simulation). (**A**) Memory usage of LAI methods in giga bytes (y-axis). (**B**) Wallclock time of the LAI methods in minutes (y-axis). The x-axis represents the number of simulated source populations in the SrcNum scenario. Each dot is the mean value of 50 repetitions of each simulations. Error bars indicate the standard deviation. ELAI, LAMP and SABER performance in memory (**A**) and time (**B**) are plotted in blue, red and yellow, respectively.

Computational performance was measured for all the analyses performed on the simulated data sets. For the DiffGenSam scenario (S=3 sources), memory consumption for the different methods ranged from 0.5Gb to 2Gb of RAM and was not highly variable across scenario variations (Figure S3). WINPOP was the fastest of the three LAI methods with a mean running time ranging from 20 s to 60 s in the DiffGenSam data sets (with three source representative populations) while SABER runs lasted between 30 min and 60 min and ELAI runs between 50 min and 4h (Figure S4). The analysis of data sets simulated under the SrcNum scenarios showed that the number of sources had the most significant impact on resource consumption (Figure 6), particularly for ELAI that used up to 10GB and 30h with S=6 sources. This corresponded to a 20-fold memory and a 38-fold computing time increases as compared with S=3 sources, (Figure 6) whereas the overall number of individuals (70 *vs.* 55) and the number of SNPs remained similar. Although memory usage increased steadily for WINPOP and SABER (from 0.7GB to 3.75GB), the computing time remained low for WINPOP (20s to 3min20s) and intermediary (up to 5h) for SABER.

## Discussion

The approaches evaluated in this study (implemented in the SABER, WINPOP and ELAI programs) were mostly developed for applications in human populations. The purpose of our study was to carry out a detailed evaluation of the accuracy of these three LAI approaches on data simulated under scenarios with features that may be encountered in studies of plant domestication or diversification involving admixture. For instance, the three methods we considered here were originally tested on data simulated by resampling haplotypes from two to three human populations in scenarios consisting of two-way or three-way admixture with up to a few tens generations post-admixture and including from 100 to 200 genotyped individuals per source representative populations in the analysis (Tang *et al.* 2006; Paşaniuc *et al.* 2009; Guan 2014).

We developed an R package (*plmgg*) to simulate genotyping data under a wider range of scenarios and sample designs that include plant-like features. Even if this simulator has some limitations (it does not simulate recombination hotspots, multiple recombination per chromosomes nor selection), it allowed us to assess the influence on LAI accuracy of the level of differentiation, of multiway admixture with up to six ancestries and of limited sampling of source populations. In addition, the impact of two plant reproduction modes was also evaluated: selfing (in a source representative population) and vegetative propagation (in the admixed population).

Overall, the two main factors that contributed to improve accuracy of all the three tested LAI approaches were the level of divergence between source populations (the higher, the better) and the number of generations since admixture (the smaller, the better) which was not surprising given their expected influence on the complexity of genome mosaics. Indeed, due to both mutations and recombination, divergence between source populations leads to increased differences among their originating haplotypes that facilitates their discrimination. Similarly, increasing the number of generations since admixture, results in shorter ancestral chromosome segment tracks, which are then more difficult to identify. However, it should be noticed that in scenarios with the most extreme level of differentiation among the source populations we considered here (τ=0.4 which corresponds to a $F_{ST}≃1-e^{-\tau }≃0.33$ in the pure-drift model of divergence we simulated), LAI accuracy remained acceptable even for the oldest admixture events (50 generations since admixture). In Citrus, average $F_{ST}$ values of 0.44 up to 0.85 were found between the four ancestral taxa depending on studies or marker types (Curk *et al.* 2015, 2016). In the cacao tree or in pineapple, pairwise $F_{ST}$ ranges between genetic groups were of 0.16 to 0.65 (Cornejo *et al.* 2018) and 0.28 to 0.94 (Chen *et al.* 2019), respectively. The lowest part of these ranges are covered in our simulations and higher values of $F_{ST}$ will actually facilitate LAI even with older admixture events. For closely related source populations, LAI approaches only performed well if admixture events were very recent (*i.e.*, below 10 generations). The three methods tested behaved roughly similarly, although WINPOP tended to be superior when source populations were more closely related whereas for more differentiated sources and between 20 and 50 generations after admixture, ELAI tended to be more accurate. This result was consistent with the WINPOP paper (Paşaniuc *et al.* 2009) that showed that WINPOP performed well with closely related populations, with its improved modeling of recombination and adaptive window length that takes into account local genetic distances between ancestral populations. As for ELAI, its two-layer HMM model helps resolving short ancestry segments that can result from increasing generation numbers after admixture (Guan 2014). In practice, differentiation among the source populations may be estimated with genotyping data available in the source representative individuals even when few individuals are available (Willing *et al.* 2012).

The timing of admixture events, required by both ELAI and WINPOP, may also represent in practice a parameter difficult to provide, especially for populations reproducing with vegetative propagation. Also, as we fixed this parameter to its true simulated value when running ELAI and WINPOP programs, our evaluation of these two methods may be overly optimistic. Yet, results obtained on the AdmxVegProp scenarios that include several generations of vegetative propagations suggests that both ELAI and WINPOP remain robust to (at least) upwardly biased estimates of the timing of admixture. In practice however, it may be valuable to check the sensitivity of the results obtained with these methods to a biologically sound range of (exponentially) varying values for this parameter. On the other hand, the timing of admixture events may also be estimated as proposed in the SABER framework. We nevertheless observed that in our settings the SABER estimations were inaccurate (see Figure S5) which suggests in turn that LAI relying on SABER is also robust to biased estimates of the timing of admixture events. Other approaches may thus be preferable to that end, for example those modeling LD decay on a whole genome basis providing sampling allows it (*e.g.*, Loh *et al.* 2013). Recently, Chen *et al.* (2019) estimated an average of 37 generations since the onset of admixture events for 22 (primarily) vegetatively propagated pineapple (var. *comosus*) hybrids, with a range of 21-55 generations.

Interestingly, we found that selfing (in a source representative population) or vegetative propagation (in the admixed population) had only a small impact on the inference accuracy. Selfing in a source population is of particular interest for banana as one of the *M. acuminata* subspecies contributing to banana hybrids is predicted to be frequently self-pollinated (Simmonds 1962). Reproduction by vegetative propagation is favored for many fruit tree crops (Miller and Gross 2011). Depending on the number of generations of sexual reproduction after admixture, vegetative propagation of admixed individuals can result in different levels of fragmentation of the mosaic structures. As mentioned above, this type of setting, with an overestimation of the generation number parameter had a minor impact on both ELAI and WINPOP, but a more notable impact on SABER inference for individuals where the overestimation was the highest.

Increasing the number of source populations (up to six tested) only marginally affected the accuracy of the tested LAI methods, particularly for ELAI. Nevertheless, this also increased the computational burden that became substantial for the ELAI program, presumably due to the higher number of model parameters. Hui *et al.* (2017) developed a tool (LAIT) to run four LAI methods including WINPOP and ELAI on a data set. They used LAIT to compare LAI methods on two-way and three-way admixture, and showed that ELAI performed better than WINPOP at the cost of increased resources consumption, which is consistent with our results.

Our results also showed that LAI methods perform similarly well for moderate to high levels of differentiation among source populations, even when the number of source representative individuals is small, which may have favorable practical consequences as it is not always possible to have access to large numbers of source representatives. Yet the three different methods behaved differently given an unbalanced data set, with a minor impact on SABER and WINPOP compared to ELAI. This may be explained by the two layers models of ELAI that ties haplotypes structure to ancestries, so that clustering will be hindered by low haplotypic variability. More generally, and in practice, assessing the number of source populations and assigning individuals to them might not be an easy task. Unsupervised clustering approaches (Pritchard *et al.* 2000; Alexander *et al.* 2009; Frichot *et al.* 2014) might be viewed as a reference choice (Stift *et al.* 2019) provided the source populations are differentiated enough and evenly represented in the data set (Puechmaille 2016). The Chromopainter method (Lawson *et al.* 2012) allows to determine ancestry sources without individuals assigned as source-representatives, provided that phased data are available.

A most critical issue regarding LAI performances was the absence of representative individuals for a given source. The results obtained on the SrcMiss simulations showed no particular bias in attributing the missing population to known ancestries. This result may come from the fact that in our simulation the population tree between the four sources is star shaped. In practice, a star shaped tree is uncommon, one known population may be closely related to the missing population and bias cannot be excluded in this case. Some empirical and specific sampling procedures have been proposed to circumvent the absence of source representatives, in the case of large proportions of unrepresented ancestry in admixed populations (Zhou *et al.* 2016). Recently, a promising and more generic alternative has been developed in the MOSAIC model of Salter-Townshend and Myers (2019) for haplotype data, which allows for extracting information on source populations from related (and possibly admixed) individuals. Yet, phased data that we purposely kept out of consideration may not be accessible for many crop species. Moreover, it has been shown that switch errors that can occur with statistical phasing (Scheet and Stephens 2006; Browning and Browning 2011) reduce LAI accuracy (*e.g.*, Guan 2014). However, haplotype-based LAI approaches such as RFMix (Maples *et al.* 2013), LOTER (Dias-Alves *et al.* 2018) and MOSAIC (Salter-Townshend and Myers 2019) that included switch error modeling demonstrated that, if properly modeled, inaccurate phasing is becoming less of a threat for LAI accuracy.

LAI on phased data may also be particularly well suited to deal with polyploidy, ploidy being highly variable in crop species (*e.g.*, pineapple 2x, cacao tree 2x, banana 2x and 3x, citrus up to 4x, sugarcane up to 12x) although statistical phasing might be challenging. Alternatively, HMM-based methods such as those proposed by (Corbett-Detig and Nielsen 2017) for Pool-Seq data may also be of value.

The evaluation of LAI methods accuracy and performance with the *plmgg* R package, showed that LAI methods are usable in the scope of crops genetics, with caution particularly in case of a missing source population. The software WINPOP seems suited when source populations are close and admixture events recent. ELAI could be particularly adapted for well differentiated and relatively well represented sources, in case of selfing in source populations, for vegetative propagation settings, and multiway admixture although for the latter, computational performance might be a limiting factor. Other parameters more specific to different plant/crop models might be evaluated using the *plmgg* package.

## References

[bib1] Alexander D. H., Novembre J., and Lange K., 2009 Fast model-based estimation of ancestry in unrelated individuals. Genome Res. 19: 1655–1664. 10.1101/gr.094052.109 19648217 PMC2752134

[bib2] Barrett, S. C. H., R. Arunkumar, and S. I. Wright, 2014 The demography and population genomics of evolutionary transitions to self-fertilization in plants. Philos. Trans. R. Soc. B Biol. Sci. 369: 20130344. 10.1098/rstb.2013.0344 PMC4071518 24958918

[bib3] Brandvain Y., Slotte T., Hazzouri K. M., Wright S. I., and Coop G., 2013 Genomic Identification of Founding Haplotypes Reveals the History of the Selfing Species Capsella rubella. PLoS Genet. 9: e1003754 10.1371/journal.pgen.1003754 24068948 PMC3772084

[bib4] Browning S. R., and Browning B. L., 2011 Haplotype phasing: existing methods and new developments. Nat. Rev. Genet. 12: 703–714. 10.1038/nrg3054 21921926 PMC3217888

[bib5] Burgarella C., Barnaud A., Kane N. A., Jankowski F., Scarcelli N., 2019 Adaptive Introgression: An Untapped Evolutionary Mechanism for Crop Adaptation. Front. Plant Sci. 10: 4 10.3389/fpls.2019.00004 30774638 PMC6367218

[bib6] Chen L.-Y., VanBuren R., Paris M., Zhou H., Zhang X., 2019 The bracteatus pineapple genome and domestication of clonally propagated crops. Nat. Genet. 51: 1549–1558. 10.1038/s41588-019-0506-8 31570895

[bib7] Christelová P., De Langhe E., Hřibová E., Čížková J., Sardos J., 2017 Molecular and cytological characterization of the global *Musa* germplasm collection provides insights into the treasure of banana diversity. Biodivers. Conserv. 26: 801–824. 10.1007/s10531-016-1273-9

[bib8] Corbett-Detig R., and Nielsen R., 2017 A Hidden Markov Model Approach for Simultaneously Estimating Local Ancestry and Admixture Time Using Next Generation Sequence Data in Samples of Arbitrary Ploidy. PLoS Genet. 13: e1006529 10.1371/journal.pgen.1006529 28045893 PMC5242547

[bib9] Cornejo, O. E., M.-C. Yee, V. Dominguez, M. Andrews, A. Sockell *et al.*, 2018 Population genomic analyses of the chocolate tree, Theobroma cacao L., provide insights into its domestication process. Commun. Biol. 1: 167. 10.1038/s42003-018-0168-6 PMC6191438 30345393

[bib10] Cornille A., Gladieux P., Smulders M. J. M., Roldán-Ruiz I., Laurens F., 2012 New Insight into the History of Domesticated Apple: Secondary Contribution of the European Wild Apple to the Genome of Cultivated Varieties. PLoS Genet. 8: e1002703 10.1371/journal.pgen.1002703 22589740 PMC3349737

[bib11] Curk F., Ancillo G., Ollitrault F., Perrier X., Jacquemoud-Collet J.-P., 2015 Nuclear Species-Diagnostic SNP Markers Mined from 454 Amplicon Sequencing Reveal Admixture Genomic Structure of Modern Citrus Varieties. PLoS One 10: e0125628 10.1371/journal.pone.0125628 25973611 PMC4431842

[bib12] Curk F., Ollitrault F., Garcia-Lor A., Luro F., Navarro L., 2016 Phylogenetic origin of limes and lemons revealed by cytoplasmic and nuclear markers. Ann. Bot. 117: 565–583. 10.1093/aob/mcw005 26944784 PMC4817432

[bib13] Danecek P., Auton A., Abecasis G., Albers C. A., Banks E., 2011 The variant call format and VCFtools. Bioinformatics 27: 2156–2158. 10.1093/bioinformatics/btr330 21653522 PMC3137218

[bib14] Dias-Alves T., Mairal J., and Blum M. G. B., 2018 Loter: A Software Package to Infer Local Ancestry for a Wide Range of Species. Mol. Biol. Evol. 35: 2318–2326. 10.1093/molbev/msy126 29931083 PMC6107063

[bib15] El Baidouri M., Murat F., Veyssiere M., Molinier M., Flores R., 2017 Reconciling the evolutionary origin of bread wheat (*Triticum aestivum*). New Phytol. 213: 1477–1486. 10.1111/nph.14113 27551821

[bib16] Falush D., Stephens M., and Pritchard J. K., 2003 Inference of population structure using multilocus genotype data: linked loci and correlated allele frequencies. Genetics 164: 1567–1587. 12930761 10.1093/genetics/164.4.1567 PMC1462648

[bib17] Frichot E., Mathieu F., Trouillon T., Bouchard G., and François O., 2014 Fast and Efficient Estimation of Individual Ancestry Coefficients. Genetics 196: 973–983. 10.1534/genetics.113.160572 24496008 PMC3982712

[bib18] Geza E., Mugo J., Mulder N. J., Wonkam A., Chimusa E. R., 2019 A comprehensive survey of models for dissecting local ancestry deconvolution in human genome. Brief. Bioinform. 20: 1709–1724. 10.1093/bib/bby044 30010715 PMC7373186

[bib19] Guan Y., 2014 Detecting structure of haplotypes and local ancestry. Genetics 196: 625–642. 10.1534/genetics.113.160697 24388880 PMC3948796

[bib20] Hellenthal G., Busby G. B. J., Band G., Wilson J. F., Capelli C., 2014 A Genetic Atlas of Human Admixture History. Science 343: 747–751. 10.1126/science.1243518 24531965 PMC4209567

[bib21] Hui D., Fang Z., Lin J., Duan Q., Li Y., 2017 LAIT: a local ancestry inference toolkit. BMC Genet. 18: 83 10.1186/s12863-017-0546-y 28877673 PMC5585928

[bib22] Lawson D. J., Hellenthal G., Myers S., and Falush D., 2012 Inference of population structure using dense haplotype data. PLoS Genet. 8: e1002453 10.1371/journal.pgen.1002453 22291602 PMC3266881

[bib23] Liu Y., Nyunoya T., Leng S., Belinsky S. A., Tesfaigzi Y., 2013 Softwares and methods for estimating genetic ancestry in human populations. Hum. Genomics 7: 1 10.1186/1479-7364-7-1 23289408 PMC3542037

[bib24] Loh P.-R., Lipson M., Patterson N., Moorjani P., Pickrell J. K., 2013 Inferring Admixture Histories of Human Populations Using Linkage Disequilibrium. Genetics 193: 1233–1254. 10.1534/genetics.112.147330 23410830 PMC3606100

[bib25] Maples B. K., Gravel S., Kenny E. E., and Bustamante C. D., 2013 RFMix: A Discriminative Modeling Approach for Rapid and Robust Local-Ancestry Inference. Am. J. Hum. Genet. 93: 278–288. 10.1016/j.ajhg.2013.06.020 23910464 PMC3738819

[bib26] Miller A. J., and Gross B. L., 2011 From forest to field: Perennial fruit crop domestication. Am. J. Bot. 98: 1389–1414. 10.3732/ajb.1000522 21865506

[bib27] Milner S. G., Jost M., Taketa S., Mazón E. R., Himmelbach A., 2019 Genebank genomics highlights the diversity of a global barley collection. Nat. Genet. 51: 319–326. 10.1038/s41588-018-0266-x 30420647

[bib28] Moreno-Estrada A., Gravel S., Zakharia F., McCauley J. L., Byrnes J. K., 2013 Reconstructing the Population Genetic History of the Caribbean. PLoS Genet. 9: e1003925 10.1371/journal.pgen.1003925 24244192 PMC3828151

[bib29] Padhukasahasram B., 2014 Inferring ancestry from population genomic data and its applications. Front. Genet. 5: 204 10.3389/fgene.2014.00204 25071832 PMC4080679

[bib30] Paşaniuc B., Sankararaman S., Kimmel G., and Halperin E., 2009 Inference of locus-specific ancestry in closely related populations. Bioinformatics 25: i213–i221. 10.1093/bioinformatics/btp197 19477991 PMC2687951

[bib31] Perrier X., Bakry F., Carreel F., Jenny C., Horry J.-P., 2009 Combining Biological Approaches to Shed Light on the Evolution of Edible Bananas. Ethnobot. Res. Appl. 7: 199 10.17348/era.7.0.199-216

[bib32] Perrier X., De Langhe E., Donohue M., Lentfer C., Vrydaghs L., 2011 Multidisciplinary perspectives on banana (*Musa spp*.) domestication. Proc. Natl. Acad. Sci. USA 108: 11311–11318. 10.1073/pnas.1102001108 21730145 PMC3136277

[bib33] Pritchard J. K., Stephens M., and Donnelly P., 2000 Inference of population structure using multilocus genotype data. Genetics 155: 945–959. 10835412 10.1093/genetics/155.2.945 PMC1461096

[bib34] Puechmaille S. J., 2016 The program structure does not reliably recover the correct population structure when sampling is uneven: subsampling and new estimators alleviate the problem. Mol. Ecol. Resour. 16: 608–627. 10.1111/1755-0998.12512 26856252

[bib35] Purcell S., Neale B., Todd-Brown K., Thomas L., Ferreira M. A. R., 2007 PLINK: A Tool Set for Whole-Genome Association and Population-Based Linkage Analyses. Am. J. Hum. Genet. 81: 559–575. 10.1086/519795 17701901 PMC1950838

[bib36] Salter-Townshend M., and Myers S., 2019 Fine-Scale Inference of Ancestry Segments Without Prior Knowledge of Admixing Groups. Genetics 212: 869–889. 10.1534/genetics.119.302139 31123038 PMC6614886

[bib37] Sankararaman S., Sridhar S., Kimmel G., and Halperin E., 2008 Estimating Local Ancestry in Admixed Populations. Am. J. Hum. Genet. 82: 290–303. 10.1016/j.ajhg.2007.09.022 18252211 PMC2664993

[bib38] Sardos J., Perrier X., Doležel J., Hřibová E., Christelová P., 2016 DArT whole genome profiling provides insights on the evolution and taxonomy of edible Banana (*Musa* spp.). Ann. Bot. 118: 1269–1278. 10.1093/aob/mcw170 27590334 PMC5155597

[bib39] Scheet P., and Stephens M., 2006 A Fast and Flexible Statistical Model for Large-Scale Population Genotype Data: Applications to Inferring Missing Genotypes and Haplotypic Phase. Am. J. Hum. Genet. 78: 629–644. 10.1086/502802 16532393 PMC1424677

[bib40] Semon M., Nielsen R., Jones M. P., and McCouch S. R., 2005 The Population Structure of African Cultivated Rice *Oryza glaberrima* (Steud.): Evidence for Elevated Levels of Linkage Disequilibrium Caused by Admixture with *O. sativa* and Ecological Adaptation. Genetics 169: 1639–1647. 10.1534/genetics.104.033175 15545652 PMC1449534

[bib41] Simmonds N. W., 1962 The Evolution of the Bananas, Longmans, London, United Kingdom.

[bib42] Staab P. R., and Metzler D., 2016 Coala: an R framework for coalescent simulation. Bioinformatics 32: 1903–1904. 10.1093/bioinformatics/btw098 27153679

[bib43] Staab P. R., Zhu S., Metzler D., and Lunter G., 2015 scrm: efficiently simulating long sequences using the approximated coalescent with recombination. Bioinformatics 31: 1680–1682. 10.1093/bioinformatics/btu861 25596205 PMC4426833

[bib44] Stift M., Kolář F., and Meirmans P. G., 2019 Structure is more robust than other clustering methods in simulated mixed-ploidy populations. Heredity 123: 429–441. 10.1038/s41437-019-0247-6 31285566 PMC6781132

[bib45] Tang H., Coram M., Wang P., Zhu X., and Risch N., 2006 Reconstructing Genetic Ancestry Blocks in Admixed Individuals. Am. J. Hum. Genet. 79: 1–12. 10.1086/504302 16773560 PMC1474129

[bib46] The 3,000 rice genomes project, 2014 The 3,000 rice genomes project. GigaScience 3: 7. 10.1186/2047-217X-3-7 PMC4035669 24872877

[bib47] The International HapMap Consortium, 2005 A haplotype map of the human genome. Nature 437: 1299–1320. 10.1038/nature04226 16255080 PMC1880871

[bib48] The Wellcome Trust Case Control Consortium, 2007 Genome-wide association study of 14,000 cases of seven common diseases and 3,000 shared controls. Nature 447: 661–678. 10.1038/nature05911 17554300 PMC2719288

[bib49] Weir B. S., and Cockerham C. C., 1984 Estimating F-Statistics for the Analysis of Population Structure. Evolution 38: 1358. 28563791 10.1111/j.1558-5646.1984.tb05657.x

[bib50] Willing E.-M., Dreyer C., and van Oosterhout C., 2012 Estimates of genetic differentiation measured by F_ST_ do not necessarily require large sample sizes when using many SNP Markers. PLoS One 7: e42649. 22905157 10.1371/journal.pone.0042649 PMC3419229

[bib51] Wu G. A., Prochnik S., Jenkins J., Salse J., Hellsten U., 2014 Sequencing of diverse mandarin, pummelo and orange genomes reveals complex history of admixture during citrus domestication. Nat. Biotechnol. 32: 656–662. 10.1038/nbt.2906 24908277 PMC4113729

[bib52] Wu G. A., Terol J., Ibanez V., López-García A., Pérez-Román E., 2018 Genomics of the origin and evolution of *Citrus*. Nature 554: 311–316. 10.1038/nature25447 29414943

[bib53] Zhao K., Wright M., Kimball J., Eizenga G., McClung A., 2010 Genomic Diversity and Introgression in *O. sativa* Reveal the Impact of Domestication and Breeding on the Rice Genome. PLoS One 5: e10780 10.1371/journal.pone.0010780 20520727 PMC2875394

[bib54] Zhou Q., Zhao L., and Guan Y., 2016 Strong Selection at MHC in Mexicans since Admixture. PLoS Genet. 12: e1005983 10.1371/journal.pgen.1005847 26863142 PMC4749250

